# CONSERVATION OF INTEGRATIVE PHYSIOLOGICAL AGING MECHANISMS ACROSS PRIMATES

**DOI:** 10.1093/geroni/igz038.025

**Published:** 2019-11-08

**Authors:** Alan A Cohen, Tina W Wey, Gabriel Dansereau, Emy Roberge, Véronique Legault, Marie Brunet, Joseph W Kemnitz, Luigi Ferrucci

**Affiliations:** 1 Université de Sherbrooke, Sherbrooke, Quebec, Canada; 2 University of Wisconsin, Madison, Wisconsin, United States; 3 NIH, Baltimore, Maryland, United States

## Abstract

Physiological dysregulation (PD) and integrated albunemia (IA) are organism-level aging mechanisms that can be measured using standard biomarkers, and in humans they have been shown to increase with age and predict health outcomes. Here, we use 10 species from the Internet Primate Aging Database (iPAD), a longitudinal database of biomarkers and mortality in captive primates, to analyze the generalizability of the role of PD and IA in aging, as well as the conservation of the underlying physiology. Human patterns are broadly but not universally replicated in primates. For example, PD increases with age in nine of eleven species, and predicts mortality in three of four. Both IA and PD can to some extent be cross-calibrated across species, indicating surprising conservation of underlying homeostatic norms; in the case of PD, the calibration weakens with phylogenetic distance.

